# MDPV (3,4-methylenedioxypyrovalerone) administered to mice during development of the central nervous system produces persistent learning and memory impairments

**DOI:** 10.1007/s43440-024-00599-0

**Published:** 2024-05-09

**Authors:** Katarzyna Kuczyńska, Katarzyna Bartkowska, Ruzanna Djavadian, Ewa Zwierzyńska, Jakub Wojcieszak

**Affiliations:** 1 Department of Pharmacodynamics, Medical University of Lodz, Muszyńskiego 1, 90-151 Łódź, Poland; 2 Laboratory of Calcium Binding Proteins, Nencki Institute of Experimental Biology Polish Academy of Sciences, 3 Pasteur St., 02-093 Warsaw, Poland

**Keywords:** MDPV, Hippocampus, Memory, Object recognition, Neurogenesis, Synaptogenesis

## Abstract

**Background:**

Synthetic cathinones (SC) constitute the second most frequently abused class of new psychoactive substances. They serve as an alternative to classic psychostimulatory drugs of abuse, such as methamphetamine, cocaine, or 3,4-methylenedioxymethamphetamine (MDMA). Despite the worldwide prevalence of SC, little is known about their long-term impact on the central nervous system. Here, we examined the effects of repeated exposure of mice during infancy, to 3,4-methylenedioxypyrovalerone (MDPV), a SC potently enhancing dopaminergic neurotransmission, on learning and memory in young adult mice.

**Methods:**

All experiments were performed on C57BL/6J male and female mice. Animals were injected with MDPV (10 or 20 mg/kg) and BrdU (bromodeoxyuridine, 25 mg/kg) during postnatal days 11–20, which is a crucial period for the development of their hippocampus. At the age of 12 weeks, mice underwent an assessment of various types of memory using a battery of behavioral tests. Afterward, their brains were removed for detection of BrdU-positive cells in the dentate gyrus of the hippocampal formation with immunohistochemistry, and for measurement of the expression of synaptic proteins, such as synaptophysin and PSD95, in the hippocampus using Western blot.

**Results:**

Exposure to MDPV resulted in impairment of spatial working memory assessed with Y-maze spontaneous alternation test, and of object recognition memory. However, no deficits in hippocampus-dependent spatial learning and memory were found using the Morris water maze paradigm. Consistently, hippocampal neurogenesis and synaptogenesis were not interrupted. All observed MDPV effects were sex-independent.

**Conclusions:**

MDPV administered repeatedly to mice during infancy causes learning and memory deficits that persist into adulthood but are not related to aberrant hippocampal development.

**Supplementary Information:**

The online version contains supplementary material available at 10.1007/s43440-024-00599-0.

## Introduction

Abuse of new psychoactive substances (NPS), as an alternative to internationally controlled drugs, poses a serious threat to public health since their appearance on the clandestine drug market at the beginning of the 21st century. Synthetic cathinones (SC) constitute the second most prevalent class of NPS, and up to date, 167 SC have been notified to the EU Early Warning System [1]. SC not only resemble the chemical structure of classic psychostimulants, such as methamphetamine or 3,4-methylenedioxymethamphetamine (MDMA), but also share their molecular mechanism of action associated with the intensification of monoaminergic neurotransmission in the brain by increasing levels of dopamine, noradrenaline, and serotonin in the synaptic cleft [2–4]. 3,4-Methylenedioxypyrovalerone (MDPV) is one of the most prominent SC that also served as a template to create a new class of SC, namely pyrovalerones [5]. MDPV produces strong and long-lasting psychostimulatory effects in humans and laboratory rodents resulting from particularly potent inhibition of dopamine and noradrenaline cellular reuptake by monoamine transporters, DAT and NET, respectively [2, 3, 6, 7].

Abuse of SC may lead to a vast array of toxic effects which mostly affect the cardiovascular and nervous system, including agitation, insomnia, shivers, seizures, hyperthermia, paresthesia, disorientation, paranoia, anxiety, hallucinations, encephalopathy, hemorrhagic stroke, coma, and even death [5, 6]. MDPV, a potent cocaine-like psychostimulant, is endowed with an exceptionally high risk of life-threatening or even fatal intoxication because its adverse effects mainly arise from the dopaminergic system stimulation [5, 6]. Although the acute toxic effects triggered by SC on the central nervous system (CNS) are widely described, the data on the long-term consequences of exposure to SC are limited. It is known that habitual use of SC may lead to dependence with withdrawal symptoms, of which the neuropsychiatric ones may be persistent, e.g. negative mood, impairment of cognitive functions [8], or working and episodic memory [9]. Furthermore, SC produce impairment of learning and memory when administered to adult [10–13] or adolescent [14–17] rodents. Gestational administration of mephedrone, a representative of SC, results in impairment of spatial and reference memory in offspring [18]. Hitherto, no research was conducted on the impact of chronic exposure to MDPV during CNS development on memory formation. Nevertheless, it was established that repeated administration of MDPV to mice between 8 and 14 days of gestation, the period when the mesolimbic system and first dopaminergic neurons are formed, causes an increase of locomotor activity of pups on postnatal day 7 and 21 [19]. Of note, the neurotoxic effects of MDPV may occur even after a single dose. At the age of 7 days, mouse pups experienced neurodegeneration of various brain regions related to memory, including the hippocampus. Intriguingly, this phenomenon did not occur in adult animals [20].

Even though the long-lasting harms caused by exposure to SC in the early period of CNS development have not been thoroughly examined, there is mounting evidence that postnatal exposure to classic psychostimulants impairs learning and memory in adolescent or adult laboratory rodents. In numerous studies, psychostimulants were administered to rodents during the critical period for hippocampus development, i.e., postnatal days 11–20 (PD11-20) [21–25], when the dentate granule cells proliferate [26]. In humans, these processes are estimated to occur during the 3rd trimester of pregnancy [26]. The treatment resulted in impairment of recognition, spatial, and reference memory manifested by decreased ability to discriminate novel objects or new localization of an object and by poor performance in the Morris or Cincinnati water maze test [21–25]. The deleterious impact of classic psychostimulants has also been documented in infants and children born to women who abused these drugs during pregnancy. Neurobehavioral changes encompass weakened response to stimuli, higher susceptibility to stress, lower intelligence quotient, attention deficits, learning difficulties, and impairments in verbal and spatial memory. Furthermore, the brains of preschool children exposed prenatally to methamphetamine have lower volume, especially with a high number of dopaminergic terminals, including the hippocampus [27, 28]. Considering the close similarity in pharmacological properties of SC to methamphetamine, the reports of SC abuse by pregnant women are of particular concern [29–31].

Therefore, the objective of the present study is to assess the effects of MDPV administered during PD11-20 on the learning and memory of adult mice of both sexes using a battery of standard behavioral tests. As the formation of new neurons and synapses in the hippocampus is crucial for learning and memory, we aimed to assess neuronal proliferation in the dentate gyrus (DG) and the expression of proteins related to synaptic plasticity in the hippocampus to elucidate the mechanism underlying memory impairments.

We hypothesized that MDPV administered to mice at PD11-20 would cause learning and memory impairments persisting into adulthood as well as it would disturb hippocampal neuroplasticity. We also aimed to determine if these impairments are sex-dependent.

## Materials and methods

### Animals

**Fig. 1:**
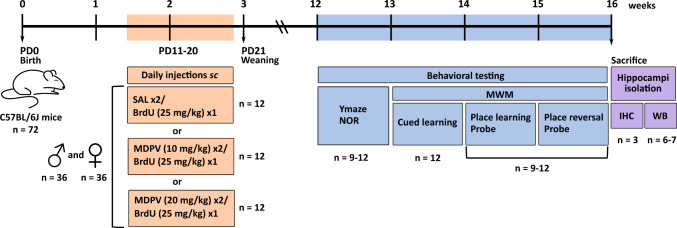
Schematic representation of treatment schedules and order of testing procedures. *BrdU* bromodeoxyuridine, *IHC* immunohistochemistry, *MWM* Morris water maze, *NOR* novel object recognition, *PD* postnatal day, *SAL* saline, *sc* subcutaneous, *WB* western blot

Experiments were performed on C57BL/6J inbred mice (Charles River, Sulzfeld, Germany). All housing conditions and experimental procedures complied with the European Directive 2010/63/EU and ARRIVE guidelines, and were approved by the Local Ethical Committee for Experiments on Animals in Łódź affiliated to Medical University of Lodz, Poland (permission no. 56/ŁB189/2020). Four male and eight female mice at the age of 8 weeks were allowed to acclimate to the conditions of the animal facility for 1 week before mating. For mating two females were placed with a male in a cage. When gestation was confirmed, pregnant females were housed singly. After weaning, mice of the same sex were housed four per cage, in standard polycarbonate cages enriched with one paper tunnel and nesting material, with free access to tap water and standard chow. Cages were kept in temperature- and humidity-controlled (20–24 °C, 45–65%), sound-attenuated rooms with automatic 12-h light/dark cycles (lights on at 6:00 a.m.). Mice from 12 litters were used for experiments. A general scheme of the experiment is presented in Fig. 1.

### Drug administration

Mice were assigned to the treatment groups according to within-litter design, meaning that male or female pups from each litter were randomly allocated to the control (saline) or treated groups (10 or 20 mg/kg MDPV). Each group consisted of 12 animals of each sex i.e., 72 mice in total. Male and female pups were treated between PD11-20. MDPV (cat #10684, Cayman Chemical, USA), solved in sterile 0.9% NaCl (Polpharma, Poland), or sterile saline was injected subcutaneously (*sc*) in a volume of 0.1 mL/10 g of body mass. MDPV was administered twice daily, at 2-h intervals, as the half-life of psychostimulants is shorter in rodents than in humans [24, 28, 32]. The selected doses of MDPV evoked pronounced behavioral effects in mice but did not induce significant lethality [20, 33, 34]. To assess neurogenesis, all mice were injected (*sc*) with BrdU (bromodeoxyuridine, cat #B5002, Sigma-Aldrich, Germany) at 25 mg/kg, once daily, 30 min after the first injection of MDPV or saline. During the administration period, pups were weighed daily. At the age of 21 days, mice were weaned. As animals reached the young adult age of 12 weeks, their learning abilities and memory were assessed using a battery of standard behavioral tests.

### Behavioral testing

All experiments were performed during the light phase (8:00–17:00). Mice were brought to the testing room 30 min before the start of each behavioral test. When relevant, after each animal apparatuses were wiped down with 70% ethanol and allowed to dry to remove olfactory cues.

#### Y-maze spontaneous alternation

The impact of MDPV on working spatial memory was assessed using the Y-maze spontaneous alternation test according to Hӧlter et al. [35] with modifications. The apparatus (Ugo Basile, Italy) was illuminated with ~ 50 lx in the center and each arm was virtually labeled A, B, and C. A tested mouse was placed in one of the arms of the Y-maze, snout facing the shorter wall, and was allowed to explore the maze freely while being recorded for 5 min. The test is based on the innate curiosity of rodents to explore an unknown environment. Mice can make correct triads consisting of entries to three different arms of the maze (e.g., ABC), or incorrect including same arm return (e.g., AAB) or alternate arm return (e.g., ABA). The number of correct alternations was scored by two experimenters blind to treatment conditions with the aid of self-developed software.

#### Novel object recognition (NOR)

Recognition memory was measured with the NOR test according to Hӧlter et al. [35] with modifications. All procedures were conducted under a dim red light. For two consecutive days, mice were singly habituated to the empty arena, a standard housing cage with an additional acrylic plate on the bottom, for 20 min. The third day started with a sample phase, in which the test mice explored the arena with two identical objects affixed to the plate (3 trials, each 5 min, with an inter-trial interval of 15 min). After 3 h retention from the last exposition to identical objects, one of the familiar objects was replaced with a novel one. After another retention interval of 24 h, a new, previously unused object was presented with the familiar one. The animals were allowed to explore objects for 5 min. The NOR task relies on an inherent motivation of mice to spend more time exploring an unfamiliar object than a previously encountered one. Object exploration was defined as sniffing or touching an object with a nose or directing a snout at the object within a small distance (< 2 cm). Sample and test sessions were recorded and analyzed by two investigators blind to treatment conditions with the aid of self-developed software. The preference index (PI) for novel objects was calculated using the following equation:

$${\text{PI}} = \frac{\text{time spent investigating novel object}}{\text{time spent investigating both objects}}\times 100\%$$

#### Morris water maze (MWM)

The MWM is a standard method for measuring hippocampus-dependent spatial learning and memory of rodents relying on a natural desire to escape from water to the ground. The test was conducted according to Vorhees and Williams [36] with modifications. A circular pool (Ø120 cm, Ugo Basile, Italy) half-filled with opaque water (20–22 °C) was virtually divided into four quadrants. The position and movement of mice were recorded with a camera mounted above the pool and analyzed in real-time with ANY-maze software (version 4.82, Stoelting, USA). The pool was located in a room providing several distant cues, with floor light intensity of ~ 100 lx. The proper test was proceeded by cued learning (lasting 3 days) to detect possible factors impairing performance, unrelated to spatial memory. The goal was a platform elevated above the water surface (approx. 1 cm) and tagged with a pole. Mice performed four trials daily, each starting randomly from one of the four predefined positions. The mouse was released into the water facing the wall and was expected to find the platform within 60 s. If the animal reached the platform it was allowed to remain on it for 15 s to observe the room. Otherwise, the mouse was manually placed on the platform for 15 s. After each trial the mouse was put for 5 min into a cage, covered with paper towels, to rest and dry. Following pre-training, the place learning (acquisition) phase (5 days, 4 trials daily) was carried out to evaluate the ability of mice to learn to swim to a specific location using environmental cues. This phase was performed according to the analogous scheme to pre-training, but the platform was submerged under the water surface (approx. 1 cm) to make it invisible to animals. On the 6th day, the platform was removed and the probe test (1 trial) assessing reference memory was conducted. Subsequently, the hidden platform was relocated to the opposing quadrant in the place reversal phase (5 days, 4 trials daily) to assess cognitive flexibility. The day after, the reversal phase was followed by the second probe test.

### Neurogenesis and synaptic plasticity assays

After the completion of behavioral experiments, separate groups of mice were used for the assessment of neurogenesis in the DG that occurred during the administration period (PD11-20), and for the measurement of proteins related to synaptic plasticity in the hippocampus.

#### Assessment of neurogenesis in the DG with immunohistochemical staining

Detection of BrdU was performed according to Bartkowska et al. [37] and Tepper et al. [38] with modifications.

Mice were anesthetized intraperitoneally with a sublethal dose of 100 mg/kg pentobarbital (Euthasol vet, Le Vet Pharma, The Netherlands) and perfused transcardially with saline followed by 4% paraformaldehyde in 0.1 M phosphate buffer (pH = 7.4). The brains were removed from the skull, post-fixed in 4% paraformaldehyde solution, cryoprotected with 30% sucrose solution, embedded in tissue freezing medium (cat #14020108926, Leica, UK), and cut into 40 µm coronal slides in a cryostat (Leica Biosystems, USA) at –20 °C. All sections of each brain were arranged into a series of ten. A randomly selected series of free-floating sections were stained immunohistochemically for BrdU. The sections were washed in 2 × SSC (saline-sodium citrate buffer), denatured in 2 N HCl for 30 min at 37 °C, and washed with 0.1 M boric acid (pH = 8.5) for 10 min. To block endogenous peroxidase, the slices were soaked for 30 min in 3% H_2_O_2_ in TBS (Tris-buffered saline). Afterward, the sections were rinsed three times for 15 min in TBS-A (TBS with 0.1% Triton X-100) and 15 min in TBS-B (TBS-A with 0.05% BSA—bovine serum albumin). After blocking for 1 h with 10% NGS (normal goat serum, cat #S26-M, Millipore, Germany) solution in TBS-B, the sections were incubated with rat anti-BrdU primary antibody (1:1000, cat #ab6326, Abcam, UK) solution in TBS-B overnight at 4 °C. Once the sections were rinsed for 15 min with TBS-A and TBS-B, they were incubated for 45 min with biotinylated goat anti-rat IgG secondary antibody (1:200, cat #112-065-003, Jackson ImmunoResearch, USA) in TBS-B. After 15-min washes with TBS-A and TBS-B, the slices were incubated for 1 h in ExtrAvidin conjugated with peroxidase (1:200, cat #E8386, Sigma-Aldrich, Germany). Peroxidase was detected by reaction with 0.05% 3,3ʹ-diaminobenzidine chromium and 0.003% H_2_O_2_ in the presence of nickel ions (cat #SK-4100, DAB Substrate Kit, Vector, USA). Finally, the slices were rinsed in TBS, applied on microscope slides, dehydrated, and coverslipped with Depex mounting medium (cat #18243.01, Serva, Germany). Images of immunostained brain sections were captured with a BX61V8 Olympus microscope with an automatic virtual slide scanning system VS120 (Olympus, Japan). The number of BrdU-labeled neurons was counted manually within the frame of 1600 × 900 μm in four representative sections of DG from both hemispheres, corresponding to the anterior (1 slice), medial (2 slices), and posterior (1 slice) regions of the DG (Supplementary material 1). The brain sections were selected across the entire DG between –1.4 and –3.4 mm to the bregma [39]. The number of BrdU-labeled cells in all sections from each mouse were averaged per animal.

#### Assessment of BrdU colocalization with immunohistochemical double-labeling

Immunolabeling of neuronal nuclear protein (NeuN) or glial fibrillary acidic protein (GFAP) colocalizing with BrdU was performed according to Bartkowska et al. [37] and Tepper et al. [38] with modifications. Some free-floating brain sections were double-immunostained for BrdU and NeuN or BrdU and GFAP to detect the fate of BrdU-labeled cells. The sections were rinsed in TBS and incubated in 10% NGS with 1% BSA in TBS-A for 1 h. Afterward, the sections were incubated with primary antibodies: rabbit anti-NeuN (1:20, cat #24307, Cell Signalling Technology, USA) or rabbit anti-GFAP (1:500, cat #Z0334, DAKO, Agilent Technologies, USA) in 5% NGS with 0,5% BSA in TBS-T overnight at 4 °C. After 30 min wash in TBS-T, the sections were incubated for 1 h with goat anti-rabbit secondary antibody conjugated with fluorochrome Alexa Fluor^®^ 568, (1:600, cat #ab175471, Abcam, UK). Eventually, the sections were permeabilized for BrdU as described above with some modifications. Shortly, the sections were consecutively incubated with 2xSSC, 2 N HCl, 0.1 M boric acid, and 10% NGS in TBS-T. Thereafter, the sections were incubated with rat anti-BrdU primary antibody (1:1000) overnight at 4 °C. The next day, biotinylated goat anti-rat IgG secondary antibody (1:100) was applied for 1 h. Then, after washing, the sections were incubated with Streptavidin conjugated with fluorochrome Alexa Fluor^®^ 488 (1:1600, cat #S11223, ThermoFisher Scientific, USA). After washing, the sections were arranged on microscope slides and coverslipped with a mounting medium (60% glycerol in PBS).

Images of double-immunolabeled BrdU and GFAP or BrdU and NeuN were captured with a confocal spinning-disk microscope (Zeiss, Germany). BrdU-labeled cells in four areas (within a square of 125.500 µm^2^) across two sections per mouse were analyzed by confocal microscopy to evaluate the colocalization of BrdU with NeuN and BrdU with GFAP cells. Then, the numbers of mature neurons (colocalization of BrdU with NeuN) and astrocytes (colocalization of BrdU with GFAP) were expressed as the percentage of BrdU-labeled cells. The percentage of double-labeled cells in all areas from each mouse was averaged per animal.

#### Measurement of proteins related to synaptic plasticity in the hippocampus by western blotting

Mice were sacrificed by cervical dislocation and their hippocampi were dissected on ice by anatomical borders, weighed, homogenized mechanically in lysis buffer supplemented with protease inhibitor cocktail (cat #04693159001, Roche, Switzerland), and centrifuged at 20,800 rpm for 45 min at 4 °C. The supernatant was collected, aliquoted, and stored at –80 °C. The samples containing 30 or 7.5 µg of protein per lane for assessment of synaptophysin (SYP) or postsynaptic density protein 95 (PSD95) expression, respectively, were loaded on 4–12% polyacrylamide NuPAGE™ Bis–Tris gel (cat #NP0321PK2, Invitrogen, USA). Proteins were separated by electrophoresis (120 V, 80 min) and electrotransferred to a nitrocellulose membrane (cat #1620112, Bio-Rad, USA; 150 mA, 1 h, 4 °C). Next, membranes were blocked in 5% milk in TBS-T (TBS with 0.2% Tween) for 60 min. The blots were incubated with rabbit anti-SYP (1:1000, cat #5461, Cell Signalling Technology, USA), rabbit anti-PSD95 (1:4000, cat #3450, Cell Signalling Technology, USA) and mouse anti-ACTB – actin beta (1:2000, cat #3700, Cell Signalling Technology, USA) primary antibodies overnight at 4 °C. On the next day, blots were washed with TBS-T (3 times, 10 min) and incubated with IRDye^®^ 800CW donkey anti-rabbit IgG (1:10,000; cat #926-32213, LI-COR Biosciences, USA,) and IRDye^®^ 680RD donkey anti-mouse IgG (1:10,000; cat #926-68072, LI-COR Biosciences, USA) secondary antibodies in 5% milk in TBS-T for 1 h at room temperature in the dark. Finally, the blots were washed with TBS-T. The intensity of protein bands was determined by measurement of fluorescence with LI-COR Odyssey DLx imager (LI-COR Biosciences, USA) and quantified with the dedicated program (Image Studio, version 5.2). The expression of SYP and PSD95 was normalized with ACTB as a loading control and showed a percentage of control from the same gel. For raw images of immunoblot membranes see Supplementary material 2.

### Data analysis

All statistical analyses were run with RStudio software (version 2022.12.0.353, Posit Software, PBC, USA). Results were fit to a linear mixed model which accounts for the effects of random factors (litter) in addition to fixed factors (treatment, sex). Afterward, two-way ANOVA (treatment ⨯ sex) was performed to determine if there was a significant effect of fixed factors and their interaction. When relevant, two-way repeated measures (RM) ANOVA (treatment ⨯ sex) was applied. Additionally, for the NOR test analysis, a one-sample t-test was applied to compare the preference index of mice with a theoretical mean of 50% indicating the random choice of an object. In all cases, *p* < 0.05 was considered significant. Differences between groups were determined using Tukey’s post hoc test. Figures were created with GraphPad Prism 6.0 (GraphPad, USA). Data are presented as mean ± standard error of the mean (SEM). Particular tests applied for each data set are provided in the result section and figure captions. As neither analysis found significant treatment ⨯ sex interaction, data from males and females were analyzed with post hoc test and presented together. Number of replicates (n) in all experiments always refers to the number of mice.

## Results

### Body mass

**Fig. 2:**
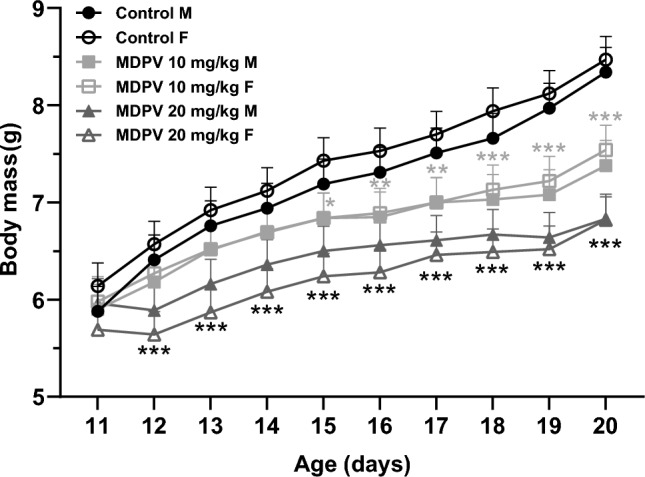
Effect of MDPV treatment during postnatal days 11–20 on body mass of mouse pups during the administration period. Data presented as mean ± standard error of the mean (SEM) (n = 12). *F* females, *M* males. *p < 0.05, **p < 0.01, ***p < 0.001 vs. control group; two-way RM ANOVA, Tukey’s post hoc test

Two-way RM ANOVA revealed that MDPV administered to mouse pups on PD11-20 caused a dose-dependent significant decrease in body mass (Fig. 2) with treatment (F_2,61_ = 34.0, *p* < 0.001), day (F_9,591_ = 249.9, *p* < 0.001) and treatment ⨯ day interaction (F_18,591_ = 13.9, *p* < 0.001) being significant factors. Sex (F_1,55_ = 0.0, *p* = 0.904) and treatment ⨯ sex interaction (F_2,60_ = 1.5, *p* = 0.229) were not significant. Tukey’s post hoc test showed that treatment with 10 mg/kg MDPV caused a decline in weight gain on PD15-20 (Fig. 2), while administration of 20 mg/kg MDPV resulted in a decrease from PD12 until the end of the treatment period (Fig. 2). Two-way ANOVA revealed that before sacrifice, there was no relevant difference in body mass between MDPV-treated groups and control animals (F_2, 54_ = 0.5, *p* = 0.626) (data not shown).

Lethality during the administration stage reached 7.69% in the control group (2 pups), 4% in the group receiving 10 mg/kg MDPV (1 pup), and 11.11% in the mice treated with 20 mg/kg MDPV (3 pups).

### Spontaneous alternation test

**Fig. 3:**
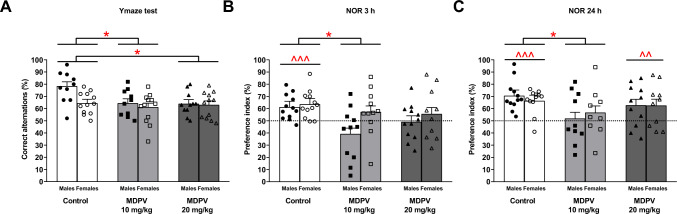
Effect of MDPV treatment during postnatal days 11–20 on the percentage of spontaneous alternations performed by mice at 12 weeks of age in the Y-maze (**A**) and the preference index in the novel object recognition (NOR) test with the 3-h interval (**B**) and with the 24-h interval (**C**). Data presented as mean ± standard error of the mean (SEM) (n = 9–12). *p < 0.05 vs. control group; two-way ANOVA, Tukey’s post hoc test. ^^*p* < 0.01, ^^^*p* < 0.001 compared to chance level using one-sample t-test

Two-way ANOVA revealed that spontaneous alternations were significantly influenced by treatment (F_2,53_ = 4.7, *p* = 0.013) and sex (F_1,59_ = 5.5, *p* = 0.023). However, no significant treatment ⨯ sex interaction (F_2,52_ = 2.6, *p* = 0.084) occurred. Tukey’s post hoc test showed that both doses of MDPV caused impairment of working spatial memory in mice. Mice from the control group performed a significantly higher percentage of correct triads of arm entries (males: 78.4%, females: 64.4%, average: 71.4%) than those treated with 10 mg/kg MDPV (males: 64.4%, females: 61.1%, average: 62.8%) and 20 mg/kg MDPV (males: 63.9%, females: 63.0%, average: 63.5%) (Fig. 3A).

### NOR test

Two way-ANOVA indicated that the percentage of time spent on exploring the novel object after the 3-h retention interval was affected by treatment (F_2,62_ = 4.5, *p* = 0.015) and sex (F_1,62_ = 4.7, *p* = 0.033), but there was no significant treatment ⨯ sex interaction (F_2,62_ = 1.4, *p* = 0.264). Tukey’s post hoc test showed that mice treated with 10 mg/kg MDPV displayed impairments in novel object recognition as their preference index averaged 48.2% (males: 39.2%, females: 57.2%) in comparison to 62.3% (males: 61.2%, females: 63.5%) of the control group. Recognition memory in mice receiving 20 mg/kg MDPV was not significantly impaired (Fig. 3B). Two-way ANOVA revealed that after the 24-h retention period interaction with the novel object was influenced by treatment (F_2,58_ = 4.1, *p* = 0.022). Sex (F_1,58_ = 0.0, *p* = 0.965) as well as treatment ⨯ sex interaction (F_2,58_ = 0.4, *p* = 0.666) were not significant factors. Tukey’s post hoc test showed that MDPV at 10 mg/kg impaired the ability of mice to discriminate novel objects from familiar ones (males: 51.8%, females: 56.7%, average: 54.3%) vs. control (males: 70.5%, females: 66.3%, average: 68.4%). The performance of mice treated with MDPV at 20 mg/kg did not vary from that of control animals (Fig. 3C). Additionally, a one-sample t-test revealed that after 3-h retention the preference index was significantly higher than 50% for control mice (t_23_ = 5.7, *p* < 0.001), while preference index of mice treated with 10 and 20 mg/kg MDPV (t_21_ = 0.4; *p* = 0.707; t_21_ = 0.6, *p* = 0.587), respectively, did not differ significantly from 50%, proving random choice of explored object and suggesting that the mice did not remember the original one (Fig. 3B). After 24-h retention the preference index significantly exceeded 50% in control mice (t_23_ = 8.0, *p* < 0.001) as well as in mice receiving 20 mg/kg MDPV (t_20_ = 3.4, *p* = 0.003). In contrast, mice treated with 10 mg/kg MDPV (t_20_ = 0.6, *p* = 0.528) divided their exploration evenly between both objects (Fig. 3C).

### Morris water maze

#### Cued learning

During pre-training all mice performed equally well in the task, showed no signs of impairment of visual and motor abilities, and learned to escape to the visible platform. Two-way RM ANOVA revealed that treatment exerted no effect on escape latency (F_2,56_ = 0.1, *p* = 0.933) and swimming speed (F_2, 58_ = 0.2, *p* = 0.850) of mice, or the total distance traveled (F_2__,__198_ = 0.0, p = 0.992) by animals. Sex did not affect escape latency (F_1,64_ = 3.2, *p* = 0.080) and swimming speed (F_1,64_ = 0.1, *p* = 0.752), however, it significantly affected total distance covered (F_2,198_ = 5.5, *p* = 0.020). In contrast, two-way RM ANOVA revealed that day was a significant factor for escape latency (F_2,132_ = 277.5, *p* < 0.001), speed (F_2,132_ = 126.9, *p* < 0.001), and total distance covered (F_2,198_ = 223.0, *p* < 0.001), indicating effective learning (data not shown).

#### Place learning

**Fig. 4:**
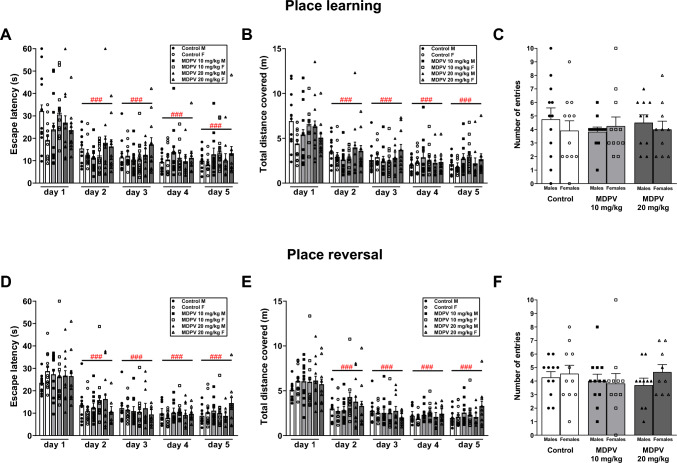
Effect of MDPV treatment during postnatal days 11–20 on performance of mice in the Moris water maze (MWM) at 14–15 weeks of age. Place learning phase: escape latency (**A**), total distance covered (**B**); probe following place learning phase: the number of entries to the former location of the platform (**C**). Place reversal phase: escape latency (**D**), total distance covered (**E**); probe following place reversal phase: the number of entries to the former location of the platform (**F**). Data presented as mean ± standard error of the mean (SEM) (n = 9–12). *F* females, *M* males. **A**, **B**, **D**, **E** ^###^p < 0.001 vs. day 1; two-way RM ANOVA, Tukey’s post hoc test. **C**, **F** two-way ANOVA

Two-way RM ANOVA revealed that treatment had no effect on escape latency (F_2,54_ = 0.9, *p* = 0.396) (Fig. 4A) and distance covered (F_2,54_ = 0.9, *p* = 0.416) (Fig. 4B). All mice, regardless of treatment, learned to reach the hidden platform because the day of the experiment was a significant factor for escape latency (F_4,248_ = 35.8, *p* < 0.001) and distance traveled (F_4,248_ = 43.9, *p* < 0.001). Tukey’s post hoc test showed that escape latency and distance traveled significantly decreased on days 2–5 compared to day 1. Sex did not affect escape latency (F_1,60_ = 0.2, *p* = 0.635) nor distance traveled (F_1,60_ = 0.5, *p* = 0.492). There was also no significant treatment ⨯ sex interaction regarding escape latency (F_2,53_ = 1.6, *p* = 0.217) and distance traveled (F_2,53_ = 1.0, *p* = 0.339).

#### Probe test following place learning

Two-way ANOVA revealed that treatment did not affect the number of entries to the former location of the platform (F_2,62_ = 0.1, *p* = 0.896) in mice (Fig. 4C). The number of entries was affected neither by sex (F_1,62_ = 0.3, *p* = 0.586) nor treatment ⨯ sex interaction (F_2,62_ = 0.5, *p* = 0.603).

#### Place reversal

Two-way RM ANOVA revealed that treatment had no significant effect on escape latency (F_2,54_ = 0.3, *p* = 0.732) (Fig. 4D), and distance traveled (F_2,54_ = 1.0, *p* = 0.392) (Fig. 4E). Day significantly affected latency to find the platform (F_4,236_ = 63.7, *p* < 0.001) and distance covered (F_4,236_ = 59.4, p < 0.001), indicating a successful learning process through the reversal phase. Tukey’s post hoc test showed that escape latency and distance traveled significantly shortened on days 2–5 compared to day 1. Sex did not affect escape latency (F_1,58_ = 0.0, *p* = 0.999) and distance traveled (F_1,58_ = 0.1, *p* = 0.748). There was no significant treatment ⨯ sex interaction in the case of escape latency (F_2,51_ = 0.1, *p* = 0.951) and distance traveled (F_2,51_ = 0.1, *p* = 0.944).

#### Probe test following place reversal

Two-way ANOVA revealed that treatment did not influence the number of entries to the previous location of the platform (F_2,55_ = 0.4, *p* = 0.691) in mice (Fig. 4F). Entries were not affected by sex (F_1,58_ = 0.7, *p* = 0.398) nor treatment ⨯ sex interaction (F_2,52_ = 0.5, *p* = 0.626).

#### Floating

During all phases of the MWM test episodes of floating, the state in which the animal remains inactive and does not move forward, occurred (Supplementary material 3). Two-way RM ANOVA revealed that during pre-training treatment had no significant impact on floating time (F_2,198_ = 0.7, *p* = 0.509). Floating time was also not affected by sex (F_1,198_ = 2.7, *p* = 0.102) nor treatment ⨯ sex interaction (F_2,198_ = 0.7, *p* = 0.509) occurred (data not shown). However, through the place learning phase, treatment affected the time of floating (F_2,60_ = 3.7, *p* = 0.031). Floating was also affected by day (F_4,264_ = 2.4, *p* = 0.046) and treatment ⨯ day interaction (F_8,264_ = 2.9, *p* = 0.004). On the other hand, sex (F_1,66_ = 0.0, *p* = 0.922) did not affect floating time and there was no interaction of treatment ⨯ sex (F_2,59_ = 0.0, *p* = 0.998). Tukey’s post hoc test showed that mice treated with 20 mg/kg MDPV remained motionless for a significantly longer time than control mice from the third to the fifth day of the place learning phase. Although two-way RM ANOVA revealed that during the place reversal phase, treatment did not significantly increase the percentage of time in which mice were floating (F_2,61_ = 2.4, *p* = 0.102), the trend of prolonged time of floating was observed in mice receiving 20 mg/kg MDPV. Sex did not affect floating time (F_1,66_ = 0.6, *p* = 0.452) and there was no treatment ⨯ sex interaction (F_2,59_ = 0.2, *p* = 0.851). To sum up, as the experiment proceeded, the duration of floating incidents showed an upward trend due to the high time of immobility occurring in some mice. Thus, the animals with a high incidence of floating, beginning from the place learning phase, were removed from further analysis to prevent obtaining potential artifacts.

### Effect of postnatal MDPV treatment on hippocampal neurogenesis and synaptic plasticity

#### Neurogenesis in the DG

**Fig. 5:**
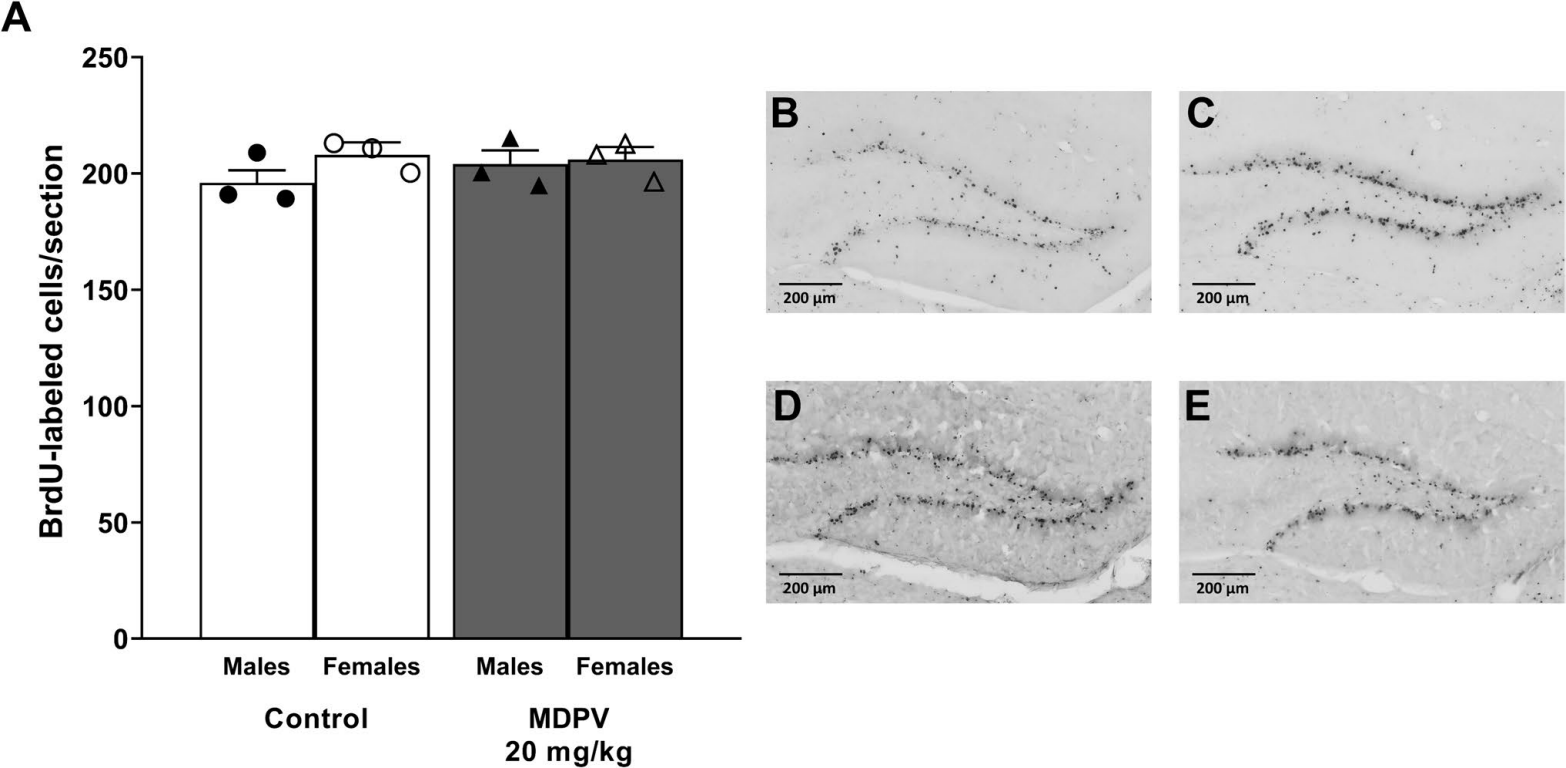
Effect of MDPV treatment during postnatal days 11–20 on proliferation and neurogenesis in the dentate gyrus (DG). Mice were treated with BrdU (bromodeoxyuridine) at postnatal days 11–20 and sacrificed at 16 weeks of age. Proliferation was assessed as the average number of BrdU-immunopositive cells counted manually in four representative sections across the entire DG per mouse (**A**). Representative images depict BrdU-labeled cells in the DG of the control males (**B**), 20 mg/kg MDPV-treated males (**C**), control females (**D**) and 20 mg/kg MDPV-treated females (**E**). Images captured at 20 × magnification under a BX61V8 Olympus microscope with an automatic virtual slide scanning system VS120 (Olympus, Japan). Data on panel (**A**) presented as mean ± standard error of the mean (SEM) (n = 3), two-way ANOVA

BrdU, a marker of the S-phase of the cell cycle, was used to study cell proliferation. BrdU injections were performed during the postnatal development of the DG in PD11-20 mice. Animals were sacrificed later, at 16 weeks of age. BrdU-labeled cells were counted separately in each DG on four representative sections and expressed as a mean number of cells per section. Two-way ANOVA revealed that during the hippocampal development period MDPV did not cause any significant differences in the number of BrdU-labeled cells in the DG (F_1,6_ = 0.3, *p* = 0.602) of mice compared to the control groups (Fig. 5A). The number of BrdU-immunostained cells was not affected by sex (F_1,6_ = 2.3, *p* = 0.178) and no treatment ⨯ sex interaction (F_1,6_ = 1.1, *p* = 0.333) occurred. As 20 mg/kg MDPV administration to pups did not affect neurogenesis in the DG, we withdrew from testing the lower (10 mg/kg) dose of MDPV.

**Fig. 6:**
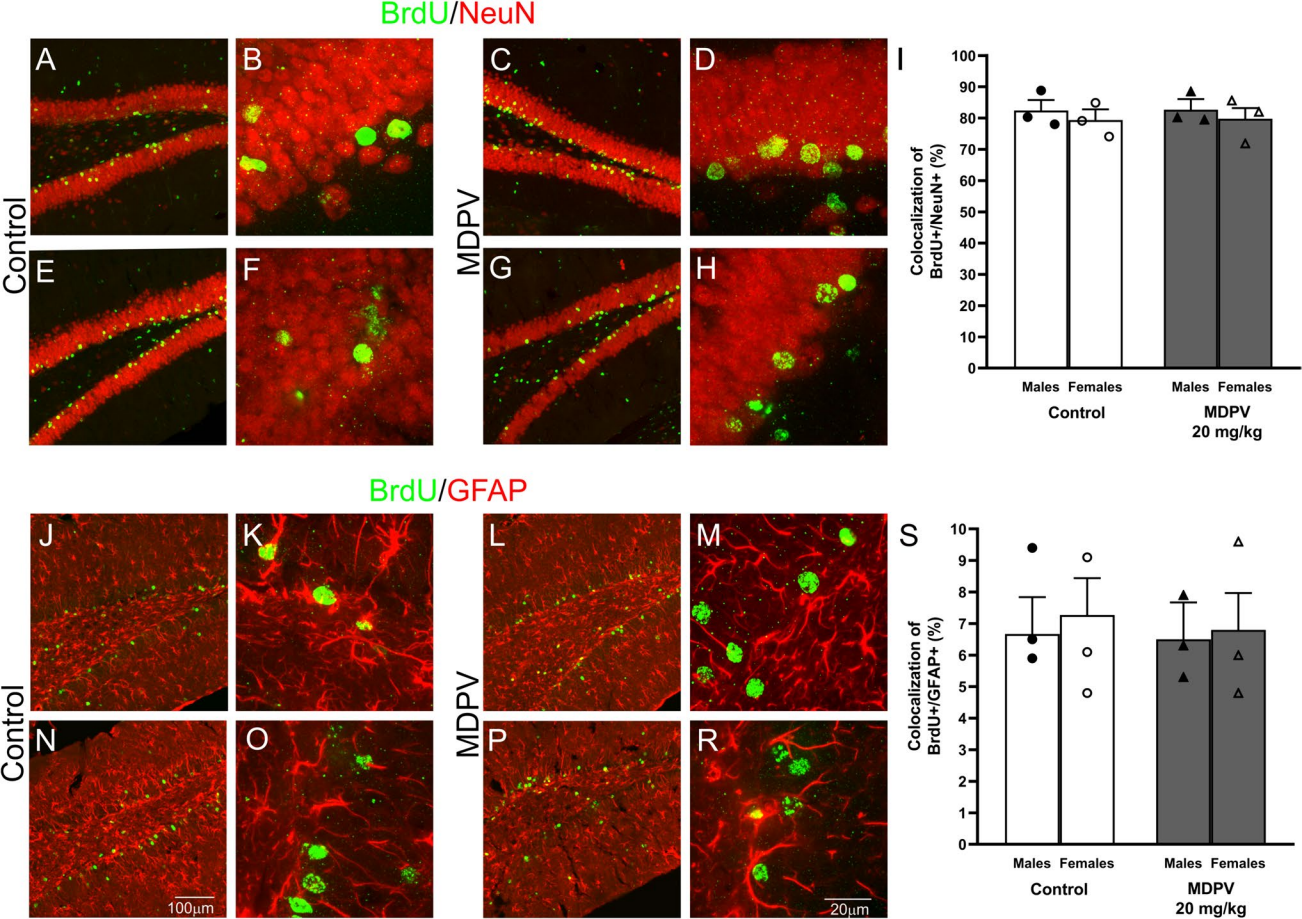
Effect of MDPV treatment during postnatal days 11–20 on colocalization of BrdU (bromodeoxyuridine) with NeuN (neuronal nuclear protein) or GFAP (glial fibrillary acidic protein) in the DG (dentate gyrus). Mice were treated with BrdU at postnatal days 11–20 and sacrificed at 16 weeks of age. Representative confocal images (**A**–**H**) showing BrdU (green) and NeuN (red) double-labeling in the DG of male (**A**, **B**) and female (**E**, **F**) controls, and 20 mg/kg MDPV-treated male (**C**, **D**) and female (**G**, **H**) mice. Colocalization of BrdU with NeuN presented as the average percentage of double-labeled BrdU + /NeuN + cells in four areas across two sections per mouse (**I**). Representative confocal images (**J**–**R**) presenting BrdU and GFAP (red) double-labeling in the DG of male (**J**, **K**) and female (**N**, **O**) control mice and 20 mg/kg MDPV-treated male (**L**, **M**) and female (**P**, **R**) mice. Colocalization of BrdU with GFAP presented as the average percentage of double-immunostained BrdU + /GFAP + cells in four areas across two sections per mouse (**S**). Images were taken at 20 × magnification (**A**, **C**, **E**, **G**, **J**, **L**, **N**, **P**) or 63 × magnification (**B**, **D**, **F**, **H**, **K**, **M**, **O**, **R**) under a confocal spinning-disk microscope (Zeiss, Germany). The scale bar in N refers to **A**, **C**, **E**, **G**, **J**, **L**, and **P**. The scale bar in **R** refers to **B**, **D**, **F**, **H**, **K**, **M**, and **O**. Data on panels (**I**, **S**) presented as mean ± standard error of the mean (SEM), (n = 3), two-way ANOVA

To test the fate of BrdU-immunopositive cells in the DG, double-immunostaining was performed using BrdU and NeuN, a neuronal marker, or BrdU and an astrocytic marker GFAP. We found that approximately 81% of BrdU-positive cells colocalized with NeuN indicating neuronal identities in the DG of both the control and MDPV-treated males and females (Fig. 6A–I). A few BrdU-positive cells were colocalized with GFAP. Almost 7% of the total BrdU-labeled cells in the DG were astrocytes (Fig. 6J–S). Two-way ANOVA revealed no significant effect of treatment on the colocalization of BrdU neither with NeuN-positive cells (F_1,8_ = 0.0, *p* = 0.905) nor with GFAP-positive cells (F_1,8_ = 0.1, *p* = 0.793) in the DG of mice. There was no effect of sex on the colocalization of BrdU with NeuN-positive cells (F_1,8_ = 0.8, *p* = 0.408), nor treatment ⨯ sex interaction (F_1,8_ = 0.0, *p* = 0.989) occurred. Sex did not affect the colocalization of BrdU with GFAP-positive cells (F_1,8_ = 0.0, *p* = 0.901), and there was no treatment ⨯ sex interaction (F_1,8_ = 0.1, *p* = 0.710).

#### Synaptic plasticity of the hippocampus

**Fig. 7:**
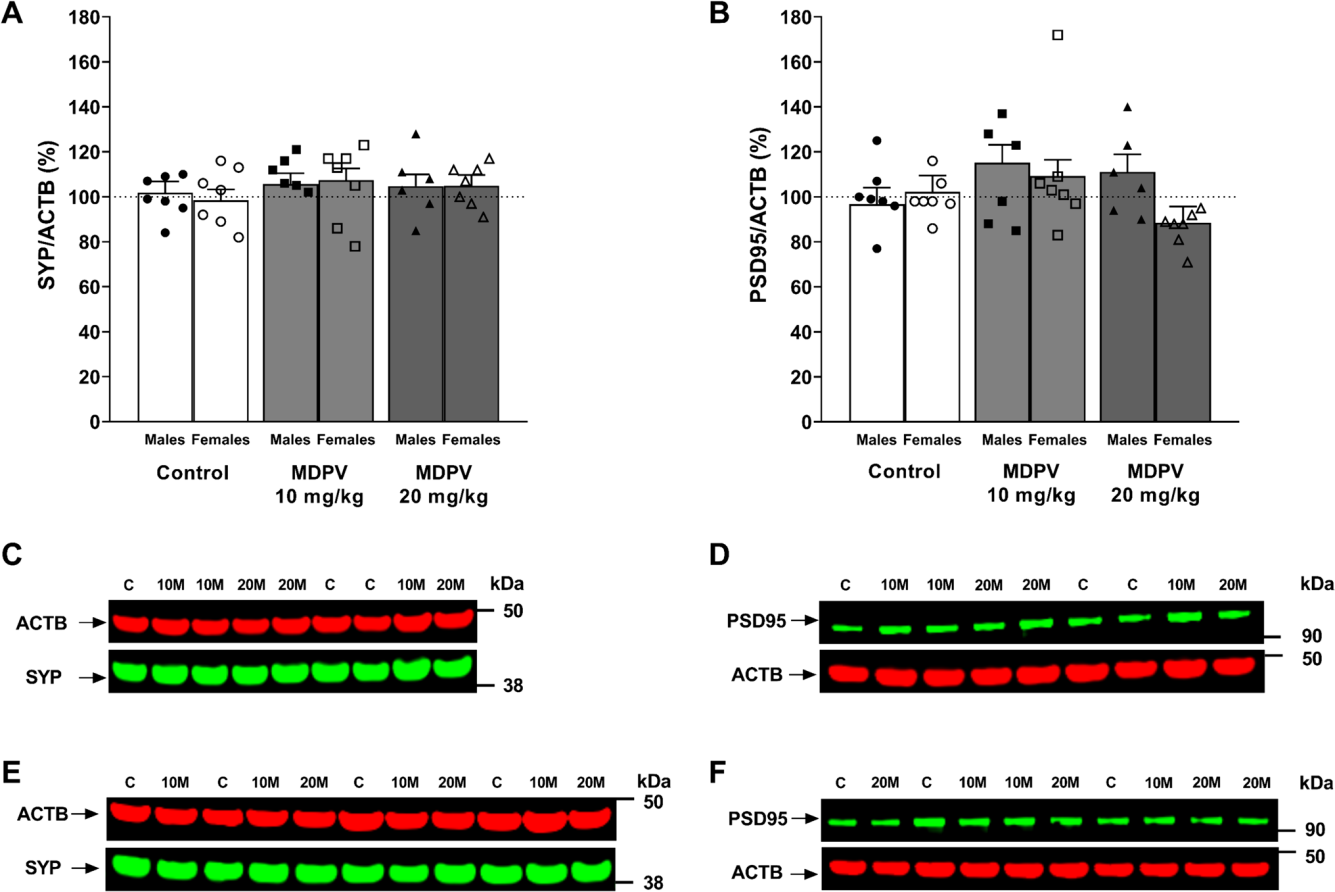
Effect of MDPV treatment during postnatal days 11–20 on expression of proteins related to synaptic plasticity in the hippocampus of mice sacrificed at 16 weeks of age: synaptophysin (SYP) (**A**) and postsynaptic density protein 95 (PSD95) (**B**) normalized to actin beta (ACTB). Representative examples of immunoblot membranes are shown for SYP in males (**C**) and females (**E**), and for PSD95 in males (**D**) and females (**F**). *C* control, *10M* 10 mg/kg MDPV, *20M* 20 mg/kg MDPV. Data on panels (**A**, **B**) presented as mean ± standard error of the mean (SEM) (n = 6–7), two-way ANOVA

To examine the plasticity of hippocampal neurons, some essential proteins implicated in synaptic regulation, particularly SYP and PSD95 proteins were studied. SYP is a presynaptic protein involved in the regulation of neurotransmitter release, while PSD95 is a postsynaptic protein that allows the detection of excitatory synapses. Two-way ANOVA revealed that MDPV did not alter the level of SYP (F_2,28_ = 1.3, *p* = 0.296) and PSD95 (F_2,28_ = 3.0, *p* = 0.068) expression in the hippocampus of mice (Fig. 7A and B). SYP expression was not affected by sex (F_1,34_ = 0.2, *p* = 0.677) nor did treatment ⨯ sex interaction (F_2,29_ = 0.1, *p* = 0.926) occur. Likewise, sex has no impact on PSD95 expression (F_1,34_ = 0.7, *p* = 0.413) and there was no treatment ⨯ sex interaction (F_2,29_ = 2.7, *p* = 0.083).

## Discussion

The present study assessed the long-lasting consequences of mice exposure to MDPV throughout the neurodevelopmental period, with a special emphasis on MDPV's impact on hippocampus functions. We have found that MDPV administered to mouse pups during PD11-20 slowed body mass gain in a dose-dependent manner. However, the reduction in body mass was not persistent. Body mass decrement is a characteristic aftermath of exposure to classic psychostimulants and SC [5, 15–17, 23, 25]. Here, we demonstrate that postnatal treatment of mice with MDPV resulted in impairment of spatial working memory as well as short- and long-term recognition memory in adulthood. We found that sensitivity to the deleterious effects of MDPV is not sex-dependent, which is not in line with some other studies performed using classic psychostimulants [21–23, 40, 41]. Impairment of learning and memory was not associated with measured markers of neuroplasticity or synaptogenesis of the hippocampus.

### Working spatial memory

It is recognized that performing tasks relying on extensive use of working memory, such as spontaneous alternation, involves activity of the medial prefrontal cortex (mPFC) connected to the hippocampus. PFC is involved in maintaining information about the immediate past, and learning the relationship between performance and results which allows the adoption of optimal behavioral strategy [42, 43]. We have found that MDPV impaired working spatial memory in mice. Currently, there is no data regarding early postnatal exposure to psychostimulants on spontaneous alternation activity. Nevertheless, our findings are in line with numerous studies conducted on mice and rats that demonstrated long-term impairment of working memory measured with Y-maze or T-maze spontaneous alternation task after binge treatment during the prenatal period or in adulthood with classic psychostimulants [44–46], khat extracts [47] as well as SC, such as mephedrone [11] or α-pyrrolidinopropiophenone (α-PPP) [48].

### Recognition memory

Recognition memory widely depends on the perirhinal cortex (PER), a structure functionally integrated with the hippocampus [49], which plays a crucial role in visual perception and discrimination of familiarity with individual objects [50]. Our results demonstrate that MDPV impaired both short- and long-term recognition memory, observed only in mice treated with the dose of 10 mg/kg. The absence of a significant effect in 20 mg/kg MDPV-treated mice may arise from high variation of results within the MDPV-exposed group. Mice treated with 20 mg/kg MDPV showed a statistically insignificant trend suggesting poor recognition memory, which is supported by a lack of significant preference for investigating the novel object after 3-h retention determined using one-sample t-test. Impairment of object recognition memory produced by MDPV is in line with previous reports, concerning classic psychostimulants [40] or SC [10, 13, 14, 17]. Repeated administration of high-dose mephedrone to adolescent rats produced impairment in NOR tested 5 weeks beyond cessation [17]. Similarly, impairments in long-term recognition memory (measured after 3-week retention) were noted in adult rats after repeated binge-like self-administration of MDPV. The authors suggested that the observed deficits in recognition memory function could be due to neurodegenerative processes detected in PER and entorhinal cortex (EC), but not in the hippocampus, which remained unaffected [13]. Likewise, Bernstein et al. [10] found that binge treatment of male Sprague–Dawley rats with MDPV impaired short-term memory in NOR tested shortly after the last injection. Moreover, MDPV leads to dopamine dysregulation in the mesocorticolimbic circuit manifested by an increase in dopamine sensibility in ventral tegmental area (VTA) and PFC [10], which receives inputs from PER, EC, and the hippocampus [51].

### Spatial learning and reference memory

Spatial and reference memory depends primarily on the hippocampal function. Learning is based on motivation, which is related to survival instinct, and on information processing consisting of acquisition, consolidation, and retrieval of optimal behavioral response which involve adult neurogenesis and synaptogenesis in the hippocampus [36, 52]. MDPV did not disturb the performance of mice in the MWM test, meaning that their spatial learning and memory were not impaired. During the MWM test, some mice tended to float during the trails instead of swimming in search of the platform. Mice were motivated to fulfill an uncomplicated task—finding a visible platform. As the testing proceeded, the tasks became progressively more demanding and mice tended to float. Since mice are adapted to inhabiting relatively dry environments, they display innate anxiety towards the aquatic environment, thus swimming in the maze is a stressful condition which may be supported by elevated corticosterone levels in mice that have undergone MWM testing [53–55]. We hypothesize that the high prevalence of floating time in mice treated with 20 mg/kg MDPV is caused by low tolerance to chronic stress experienced throughout the experiment which manifested by a gradual decrease of motivation, understood as apathy and diminishment of goal-directed behavior [56]. Further studies are needed to determine MDPV impact on stress and motivation.

The fact that MDPV did not impair spatial memory is not in line with some previous reports on the effects of perinatal administration of methamphetamine or MDMA on memory of adult animals [21, 24, 25, 57]. By contrast, there are papers suggesting no changes in spatial learning and reference memory after prenatal exposure to methamphetamine (e.g., [58]). To date, only a few studies on SC have been performed. Maternal exposure to mephedrone impaired spatial learning and reference memory of the offspring mice at the age of 60 days [18]. Similarly, mephedrone worsened the learning and probe performance of adolescent mice one week after cessation [59]. Results obtained by López-Arnau et al. [15, 16] are somehow consistent with our data. Although these studies showed that mephedrone- or methylone-treated adolescent male rats displayed an impairment of the reference memory in the MWM tested one week after drug exposure, the spatial learning process was preserved [15, 16]. Likewise, eight weeks after abruption from the binge-like regimen of mephedrone and methylone, den Hollander et al. [11] did not detect deficits in the learning process and recall trial in mice, whereas in the probe trial of the reversal phase of the MWM, their performance improved [11].

Numerous studies have shown that the formation of new neurons and synapses in the hippocampus is crucial for spatial learning and memory [36]. The majority of neurons within the DG of the hippocampal formation are produced after birth, and approximately 85% of granule cells that constitute the granular layer of the DG, are generated postnatally [60]. Our study revealed that MDPV did not impair neurogenesis in the DG of pups during early postnatal life as we detected no alternation in the number of BrdU-labeled as well as BrdU+/NeuN+ cells in the DG between control and MDPV-treated groups. Furthermore, memory formation is facilitated via the plasticity of synaptic connections dependent upon specific neural activity and protein synthesis [61]. Adult-born granule neurons need to integrate into hippocampal circuitry by establishing synaptic connections, whereas in preexisting neurons synaptic generation and potentiation occur. The number of synapses can be estimated by measuring the expression of synaptic markers of neuronal connectivity [62]. SYP constitutes a major protein of synaptic vesicle membrane, present abundantly in presynaptic endings of the CNS, where it is involved in synapse formation and transmission [63]. PSD95 is a postsynaptic protein, responsible for morphological maturation of adult-born neurons and for the formation of dendritic spines [62]. Of notion, dopamine plays a vital role in the regulation of long-term plasticity in the hippocampus, enhancing learning and memory [61]. We found that MDPV did not alter the expression of either SYP or PSD95 in mice hippocampus suggesting that synaptoplasticity in adult mice was not severely affected, which is supported by behavioral results.

Given that the hippocampus is the pivotal brain structure responsible for various cognitive functions, our main objective was to determine whether MDPV disrupts its function. Some reports indicated that psychostimulants can impede types of memory that not only rely on the hippocampus but also on other brain regions functionally connected to the hippocampus, e.g., EC contributing along with the hippocampus to allocentric navigation, while egocentric navigation in Cincinnati water maze test is striatum-dependent [64]. All things considered, we have adapted a complex approach to detect a broad range of learning and memory impairments with behavioral tests not only typically assessing the functioning of the hippocampus, but also of structures adjacent and interconnected with it. Surprisingly, we did not detect impairments in spatial learning and memory. This observation is supported by the results of molecular analysis showing no morphological and functional disturbances in hippocampal development.

We also determined that the effects of MDPV on learning and memory were sex-independent. The majority of experiments assessing harmful effects of psychostimulants on memory of rodents were performed on males solely [11–13, 15–17, 44–46, 48, 58, 59]. Those involving both sexes usually suggest that females are more vulnerable to the neurotoxic effects of classic psychostimulants [21–23, 41]. For instance, prenatally cocaine-exposed adult female rats displayed spatial learning and memory impairment in a hippocampal-dependent eight-arm radial maze test but the memory of males remained intact [41]. Likewise, spatial memory of adult rats treated with cocaine during PD11-20 appeared to be more impaired in females [22]. Moreover, only females exposed to methamphetamine during PD11-20 showed impairments in ability to locate hidden platform in the MWM in adulthood [21, 25]. Nevertheless, it was also reported that some memory deficits occurred in a sex-independent manner as mice of both sexes exposed to methamphetamine during hippocampal development showed novel object recognition impairments in adulthood [21, 23] and adolescence [65]. On the other hand, some studies suggested a higher susceptibility of males to detrimental effects evoked by psychostimulant drugs of abuse on memory [40]. Dong et al. [40] observed that only male offspring of dams exposed to methamphetamine from adolescence to adulthood, including pregnancy, suffered from impairment of short-term memory recognition in the NOR test and long-term spatial memory retention in MWM. In conclusion, it should be noted that preclinical studies have their shortcomings such as experimental protocols, different statistical approaches or small sample size that may lead to disparate results.

The present study also has its limitations. The disparity between the results of the present study and some previous reports may be accounted for the subtle differences in the experimental design. In the MWM test differences are an unavoidable consequence of a low degree of standardization of protocols. Other factors that markedly varied across studies were psychostimulant compounds, doses used, dosing regimens, and age while treating animals. In the vast majority of studies on rodents, treated within the key window of hippocampus development, methamphetamine was administered. MDPV inhibits with extraordinarily high potency and selectivity DAT. It is also a potent inhibitor of NET. Moreover, in contrast to methamphetamine, MDPV does not act as a substrate for monoamine transporters [2]. Although both drugs increase dopaminergic and noradrenergic neurotransmission, different mechanisms of action may be the reason why the results obtained with methamphetamine were not reproduced with MDPV. On the other hand, mephedrone has a nearly equipotent affinity for DAT and serotonin transporter (SERT), while methylone exhibits a predominant affinity towards SERT. Moreover, both compounds act not only as uptake inhibitors but also as monoamine transporter substrates [2, 3], which may be the cause of some differences in outcomes in the MWM.

Importantly, only a few papers on the effects of SC on the developing brain have been published so far. To our knowledge, the current study is the first attempt to assess the effects of MDPV administered during PD11-20 on the learning and memory of adult mice of both sexes. Further research needs to be conducted to explore MDPV-induced neurocognitive impairments observed in the present study.

## Conclusions

The current study demonstrates that MDPV administered to mice during the development of the CNS (PD11-20) induces learning and memory deficits in young adult animals. Postnatal exposure to MDPV impaired working spatial memory and recognition memory manifested by poor performance in spontaneous alternation and NOR test, respectively. However, MDPV did not impair hippocampal-dependent spatial memory and learning in the MWM test, but it prolonged the duration of immobility which is presumably linked to a decline in escape motivation. It should be emphasized that the impact of MDPV on animal motivation remains an important underexplored research venue. Taken together, the results of the behavioral studies indicate that the memory deficits did not arise from disruption of hippocampal functions. This observation was confirmed by unaltered neurogenesis in the DG during the administration period as well as synaptogenesis in the hippocampus of adult mice. All behavioral effects of MDPV were sex-independent. In light of our findings, we conclude that exposure to MDPV during the CNS development period poses a substantial risk of learning and memory impairments occurring in adulthood.

### Supplementary Information

Below is the link to the electronic supplementary material.

Supplementary file1 (TIF 420682 KB)

Supplementary file2 (PDF 506 KB)

Supplementary file3 (TIF 3167 KB)

## Data Availability

The datasets generated during and/or analyzed during the current study are available from the corresponding author upon reasonable request.

## Abbreviations

ACTB: Actin beta
BSA: Bovine serum albumin
BrdU: Bromodeoxyuridine
CNS: Central nervous system
DAT: Dopamine transporter
DG: Dentate gyrus
EC: Entorhinal cortex
GFAP: Glial fibrillary acidic protein
MDPV: 3,4-Methylenedioxypyrovalerone
MDMA: 3,4-Methylenedioxymethamphetamine
MWM: Morris water maze
NET: Norepinephrine (noradrenaline) transporter
NeuN: Neuronal nuclear protein
NGS: Normal goat serum
NOR: Novel object recognition
NPS: New psychoactive substances
PD: Postnatal day
PER: Perirhinal cortex
PFC: Prefrontal cortex
PSD95: Postsynaptic density protein 95
SC: Synthetic cathinones
SERT: Serotonin transporter
SSC: Saline-sodium citrate buffer
SYP: Synaptophysin
TBS: Tris-buffered saline
