# Supplementary material for: MDPV (3,4-methylenedioxypyrovalerone) administered to mice during development of the central nervous system produces persistent learning and memory impairments
Source: Pharmacol Rep. 2024 May 9;76(3):519–34. doi: 10.1007/s43440-024-00599-0 (PMC11126454; doi:10.1007/s43440-024-00599-0)
Supplement: Supplementary file 2 — Supplementary file2 (PDF 506 KB) [file 43440_2024_599_MOESM2_ESM.pdf]

1A

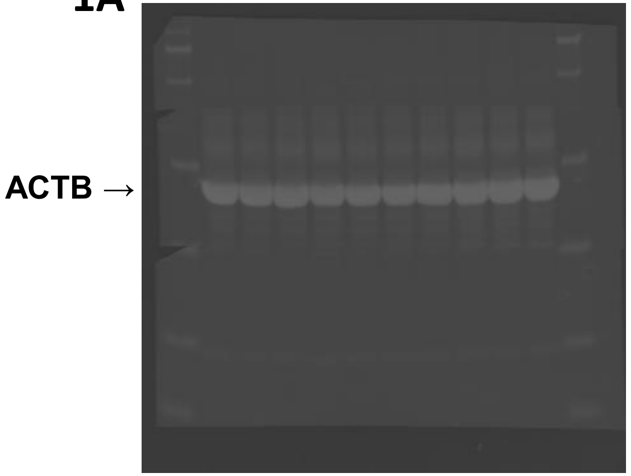

1B

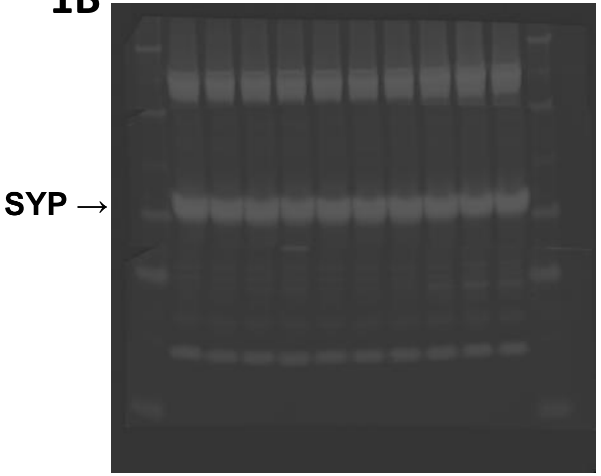

2A

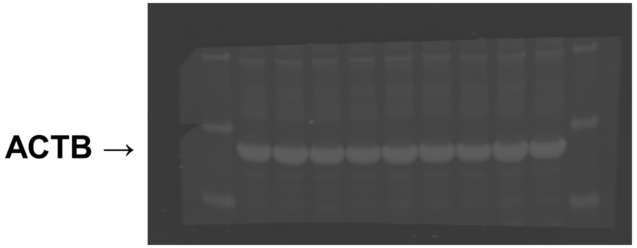

2B

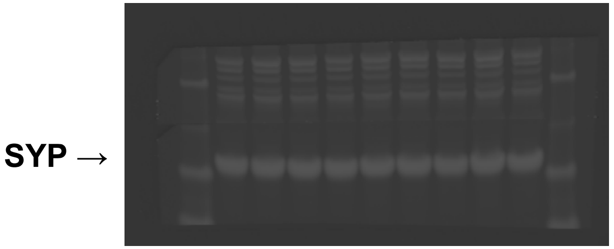

3A

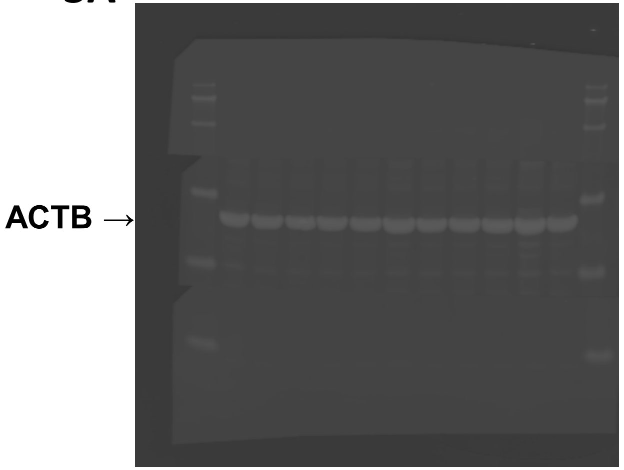

3B

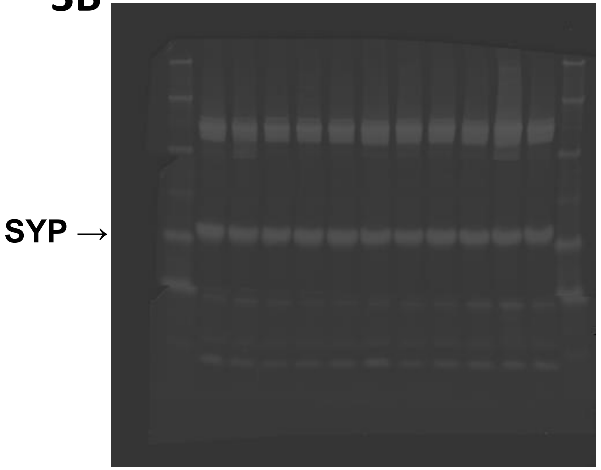

4A

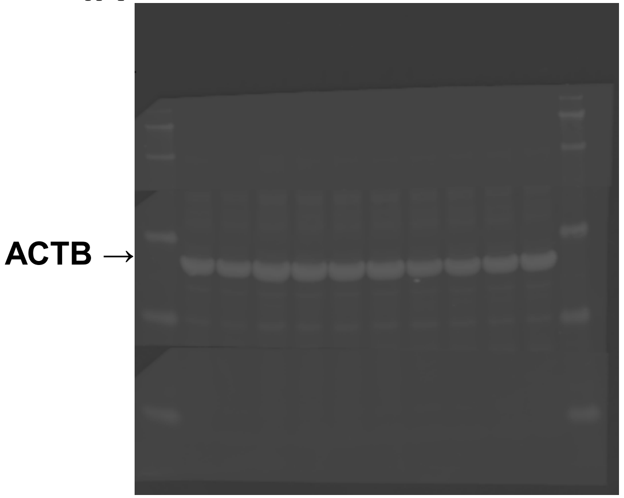

4B

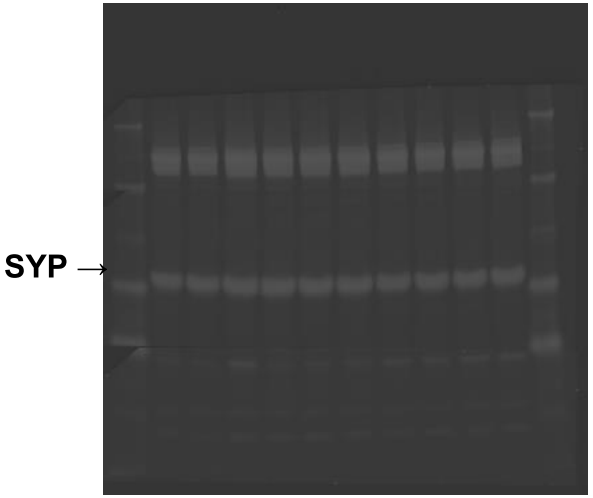

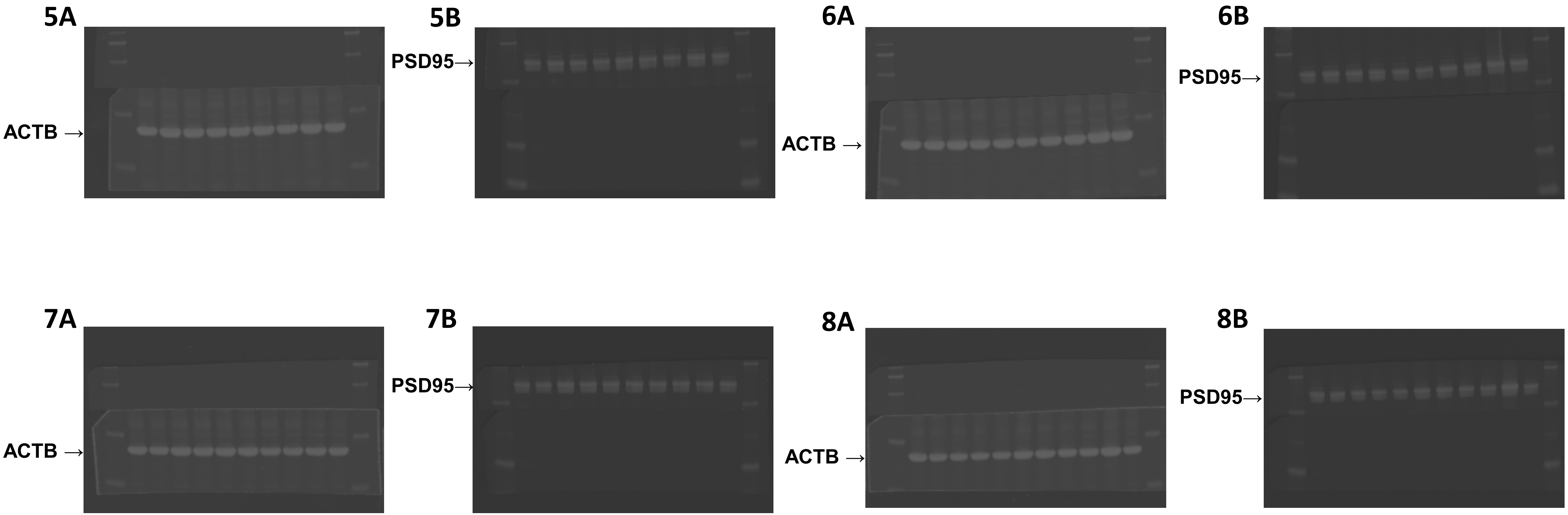

Images of raw unprocessed immunoblot membranes used in the statistical analysis of the expression of synaptophysin (SYP) and postsynaptic density protein (PSD95) in the hippocampus of mice treated during postnatal days 11-20 with saline or MDPV (10 or 20 mg/kg) . The intensity of protein bands was determined by measurement of fluorescence with LI-COR Odyssey DLx imager (LI-COR Biosciences, USA). Only indicated rows from each membrane were analyzed. The expression of SYP and PSD95 was normalized with actin beta (ACTB). Membranes labeled with the same number are the same membranes captured in the 700 nm channel (A) to detect ACTB (1A, 2A, 3A, 4A, 5A, 6A, 7A, 8A) and in the 800 nm channel (B) to visualize SYP (1B, 2B, 3B, 4B) or PSD95 (5B, 6B, 7B, 8B). Immunoblot membranes 2A,B, 3A,B for SYP and 5A,B, 7A,B for PSD95 are shown in the main manuscript.
